# Estrogen Receptor Gene 1 (*ESR1*) Mediates Lipid Metabolism in Goose Hierarchical Granulosa Cells Rather than in Pre-Hierarchical Granulosa Cells

**DOI:** 10.3390/biology12070962

**Published:** 2023-07-05

**Authors:** Qingyuan Ouyang, Hengli Xie, Mingxia Ran, Xi Zhang, Zhiyu He, Yueyue Lin, Shenqiang Hu, Jiwei Hu, Hua He, Liang Li, Hehe Liu, Jiwen Wang

**Affiliations:** Farm Animal Genetic Resources Exploration and Innovation Key Laboratory of Sichuan Province, Sichuan Agricultural University, Chengdu 611130, China; oyqy222@163.com (Q.O.); xiehengli2022@163.com (H.X.); 18227585649@163.com (M.R.); zhangx802802@163.com (X.Z.); zhiyuhesicau@163.com (Z.H.); lyy3078539326@163.com (Y.L.); shenqiang.hu@sicau.edu.cn (S.H.); hujiwei1990@126.com (J.H.); hehua023@126.com (H.H.); cnliliang@foxmail.com (L.L.); liuee1985@sicau.edu.cn (H.L.)

**Keywords:** ESR1, goose, granulosa cells, lipid metabolism

## Abstract

**Simple Summary:**

Our study successfully cloned and obtained an 1866 bp segment of the full-length coding sequence (CDS) region of the estrogen receptor gene 1 (*ESR1*) in Sichuan white geese. We compared the involvement of *ESR1* in lipid metabolism between pre-hierarchical granulosa cells and hierarchical granulosa cells in geese. Our findings indicate that *ESR1* plays a more significant role in the lipid metabolism of hierarchical granulosa cells. When *ESR1* is overexpressed in hierarchical granulosa cells, it leads to a reduction in lipid droplet deposition, cholesterol, and triglycerides, primarily regulated by *APOB* and *PPARα*, which control lipoprotein synthesis. Conversely, interfering with *ESR1* in hierarchical granulosa cells results in increased lipid droplet deposition, cholesterol, and triglycerides. This increase is jointly mediated by *ACCα, DGAT1*, and *SCD*, which regulate fatty acid synthesis, as well as *CPT1* and *ATGL*, which are involved in fatty acid degradation.

**Abstract:**

(1) Background: The role of estrogen receptor gene 1 (*ESR1*) in female reproduction and lipid metabolism has been extensively investigated. However, its contribution to lipid metabolism during the development of poultry follicles remains unclear. (2) Methods: This study aimed to explore the function of *ESR1* via overexpressing (*ESR1*^ov^) and interfering (*ESR1*^si^) with its expression in pre-hierarchical granulosa cells (phGCs) and hierarchical granulosa cells (poGCs). (3) Results: We successfully cloned and obtained an 1866 bp segment of the full-length CDS region of the Sichuan white goose *ESR1* gene. In phGCs of the *ESR1*^ov^ and *ESR1*^si^ groups, there were no significant changes compared to the control group. However, in poGCs, the *ESR1*^ov^ group exhibited decreased lipid deposition, triglycerides, and cholesterol compared to the control group, while the *ESR1*^si^ group showed increased lipid deposition, triglycerides, and cholesterol. The expression of *APOB* and *PPARα* was significantly reduced in the *ESR1*^ov^ group compared to the *ESR1*^ov^-NC group. Moreover, significant changes in the expression of *ACCα, DGAT1, SCD, CPT1*, and *ATGL* were observed between the ESR1^si^ and ESR1^si^-NC group. (4) Conclusions: These findings shed light on the function and molecular mechanism of *ESR1* in lipid metabolism in goose poGCs, providing a better understanding of the physiological process of goose follicular development.

## 1. Introduction

The annual egg production of geese ranges from 20 to 60, and enhancing egg production is a major focus of breeding research. The egg laying performance of poultry relies on the proper maintenance of follicle selection, development, and maturation. Granulosa cells, being the primary component of the follicle wall [1], have been increasingly recognized for their critical role in various stages of follicle development. During follicle selection, development, and maturation, there is a significant accumulation of lipids, and lipid metabolism has been identified as a vital regulatory mechanism [2,3]. These studies indicate that regulating lipid metabolism in granulosa cells is a key step in follicle selection, development, and maturation. Therefore, gaining insights into the molecular mechanism that govern lipid metabolism in granulosa cells during the development of goose egg follicles can provide a valuable theoretical reference for enhancing goose egg production.

As a well-established regulator of female reproduction, the estrogen receptor gene *ESR1* plays a crucial role in this process. Within the first intron of *ESR1,* there exists a single nucleotide mutation site known as ESR1-PvuII, which directly affects the transcription of estrogen response elements and subsequently the estrogen pathway [4,5]. Numerous mouse experiments utilizing gene editing technology have provided substantial evidence highlighting the significance of ESR1 in the development and maintenance of ovarian function [6,7,8]. More recently, investigations have emerged establishing a connection between *ESR1* and follicle development, along with its impact on poultry egg production [9,10]. Furthermore, the disruption of *ESR1* function in humans [11,12] and mutant rodent models [13,14] has been observed to result in obesity and metabolic dysfunction.

Although the role of *ESR1* in lipid metabolism and reproduction has been extensively studied, there is a lack of research confirming its involvement in lipid deposition during poultry follicle development. Therefore, the objective of this study was to clone and obtain the sequence of goose *ESR1* and investigate its expression in granulosa cells at different stages of follicle development. Additionally, we aimed to elucidate the impact and molecular mechanism of *ESR1* on lipid metabolism during follicle development stage by manipulating *ESR1* expression through overexpression and interference in goose granulosa cells (both pre-hierarchical and hierarchical). The findings from this study have the potential to provide valuable theoretical insights for enhancing goose egg production.

## 2. Materials and Methods

### 2.1. Sample Collection

Ethical approval on animal survival was provided by the Animal Welfare and Ethics Committee of the Institute of Animal Sciences (IAS), Sichuan Agricultural University: Approval No. 20180034. The samples used in this study consisted of laying geese obtained from the Waterfowl Breeding Laboratory of Sichuan Agricultural University (Ya’an, Sichuan, China). These geese were healthy Sichuan white geese from the same incubation batch, reared in identical environmental conditions, and at the same peak of laying concerning weight and time. Euthanasia of the geese was performed by neck exsanguination, following which their entire ovaries were extracted and placed in preheated PBS buffer solution at 37 °C. The follicular granular layers were subsequently stripped using the method described by Gilbert et al. [15]. The granular layers from three goose were used to obtained the *ESR1* expression in different follicle development (small yellow follicle (SYF), large yellow follicle (LYF), F5, F4–2, F1). In the cell experiment, the ovarian follicles were divided into two classes: pre-hierarchical (6 to 8 mm and 8 to 10 mm in diameter) and hierarchical (F5–F2, F2 > F3 > F4 > F5) based on their diameter.

### 2.2. Transcriptome Analysis

Transcriptome sequencing data of granular layers of follicles in Sichuan white goose and Tianfu meat goose at different developmental stages were downloaded from previous studies: NCBI PRJNA506334 [16] and PRJNA552525 [17]. The data underwent quality control using the FastaQC software, and low-quality reads were filtered out to obtain clean reads. These clean reads were then aligned to our assembled Sichuan white goose genome using HISAT2 software (version 2.2.1) [18]. The resulting SAM file was converted to a BAM file and sorted using SAMtools (version 1.10). Subsequently, the expression level of each transcript was calculated and the counts of *ESR1* extracted through featureCounts (version 1.6.0) [19]. To visualize the expression level of ESR1 (in terms of read counts), Prism 9.0 Graphpad software was employed.

### 2.3. Cloning CDS Region of Goose ESR1

The *ESR1* mRNA reference sequence was extracted from the reference genome of Sichuan white geese. Primers for amplifying the full-length CDS region were designed using Primer Premier 5.0 software: Forward primer: CCGAAGTAATGGCAACAACCT; Reverse primer: TCCCACTCAGGAAGATACCAATA. Total RNA was extracted from the granular layers using the TRIzol^®^ Reagent (Plant RNA Purification Reagent for plant tissue) following the manufacturer’s instructions (Invitrogen, Carlsbad, CA, USA), and genomic DNA was removed using DNase I (TaKaRa, Dalian, China). The quality of the extracted total RNA was assessed using the 2100 Bioanalyzer (Agilent Technologies, Santa Clara, CA, USA), and the concentration of total RNA was determined using the ND-2000 (NanoDrop Technologies, Wilmington, DC, USA). Only RNA samples meeting the following criteria were used for cDNA synthesis: OD260/280 = 1.8~2.2, OD260/230 ≥ 2.0, RIN ≥ 6.5. cDNA was synthesized using the PrimeScript RTTM Reagent Kit (TaKaRa, Dalian, China). PCR amplification was performed using cDNA as template with the following reaction mixture: 1 µL forward primer (10 μmol/L), 1 µL reverse primer (10 μmol/L), 1 µL cDNA, 12.5 µL 2 × Rapid Taq Master Mix, 9.5 µL ddH_2_O. The procedure was as follows: 95 °C for 3 min; 35 cycle for “95 °C for 15 s, 60 °C for 15 s, 72 °C for 27 s”; 72 °C for 5 min. The amplified product was analyzed by agarose gel electrophoresis (1.5% agarose gel), and the target fragment was extracted and purified using the FastPure Gel DNA Extraction Mini Kit (Vazyme, Nanjing, China). The amplified product was ligated to a vector using the 5 min TA/Blunt-Zero Cloning Kit (Vazyme, Nanjing, China) and then transformed using DH5α competent cells. The transformed products were added to the liquid medium without ambenzyl and cultured in a gas bath thermostatic oscillator for 1 h, and then they were centrifuged at 4000× *g* speed for 10 min. The supernatant (700 µL) was discarded, and the remaining liquid and precipitates were mixed and transferred to solid LB medium plates. The uniformly coated plates were placed in an electrothermal incubator at 37 °C for 12 h. A single colony was selected and incubated in 1 mL LB medium containing 0.1% ampicillin for 5 h oscillating at 37 °C. The bacterial solution was used as a template for PCR amplification using M13F (GTTGTAAAACGACGGCCAG) and M13R (CAGGAAACAGCTATGAC) primers. The PCR system and procedure were the same as described above. The amplified products were sent to Sangong (Shanghai, China) for Sanger sequencing.

### 2.4. Sequence Analysis of Goose ESR1

*ESR1* gene sequences of species such as *Gallus gallus* (NM_205183.2), *Anas platyrhynchos* (XM_021275842.3), *Homo sapiens* (NM_000125.4), *Mus musculus* (NM_001302531.1), *Xenopus Laevis* (XM_041562111.1), and *Danio rerio* (NM_152959.1) were downloaded from NCBI. The DNAMAN software package was utilized to convert the sequenced CDS region sequence into an amino acid sequence. The nucleotide sequences of ESR1 amino acids were aligned using the Cluster omega tool (http://www.ebi.ac.uk/Tools/msa/clustalo/, accessed on 29 October 2022), and the DNAMAN software package was employed to generate multiple sequence alignment images. The evolutionary tree was constructed using MEGA X software through the Maximum Likelihood method with a bootstrap value of 1000, and the data visualization was performed using the Figtree software package. The amino acid sequence of ESR1 underwent domain prediction using the CCD online tool (http://www.ncbi.nlm.nih.gov/Structure/cdd/cdd.shtml, accessed on 30 October 2022).

### 2.5. Isolation and Culture of phGCs and poGCs

The granular layers were collected from follicles and cut into small pieces using scissors in a 5 mL centrifuge tube. The mixture was then centrifuged for 5–10 min until no obvious precipitation was observed. The resulting mixture was transferred to a 15 mL centrifuge tube containing 0.3% type II collagenase for digestion. During digestion, the mixture was placed in a water bath at 37 °C and oscillated for about 3 min until the granulosa cells were completely dispersed. The digestion was terminated with cold PBS, and the scattered cells were filtered through a 200-mesh sieve and separated by centrifugation at 1000 RPM for 10 min. The cell morphology was observed under an Olympus microscope. Finally, the cell density was measured, and the cells were cultured in DMEM/F12 medium supplemented with 10% FBS. To prepare the medium, 50 mL of FBS was filtered using a 0.22 µM disposable filter and added to 450 mL of DMEM/F12. Then, 5 mL of penicillin-streptomycin mixture was added to the medium, mixed, and stored at 4 °C. The cells were inoculated into the culture plate at a density of 4 × 10^5^/mL and transferred to a carbon dioxide constant temperature incubator for culture. After 6 h of culture, the medium was changed to remove the non-adherent cells. The cell morphology was recorded using a microscope at 48 and 96 h.

### 2.6. Construction of ESR1 Overexpression and Interference Model in GCs

The previously cloned CDS region sequence of *ESR1* was sent to Sangong (Shanghai, China) and Gima Biotechnology Co., Ltd. (Shanghai, China) for the design and synthesis of overexpression vector (pcDNA3.1) and siRNA, as shown in Table 1. After transfection for 24 h, cell samples were collected following a media change. Then, the optimal concentration was then used to transfect ESR1-siRNA and siRNA-NC, respectively, and cell samples were collected 24 h after transfection. RNA extraction and cDNA synthesis were performed on the collected cell samples using the aforementioned method. The expression of *ESR1* was detected using the previously described method.

### 2.7. Oil Red O Staining

A 0.5% Oil Red O solution was prepared using isopropanol. Subsequently, the 0.5% Oil Red O solution was diluted with PBS in a 2:3 ratio to obtain a 0.3% Oil Red O solution. The 0.3% Oil Red O solution was then filtered using double-layer filter paper. The cells to be stained were washed three times with PBS and fixed at room temperature with 4% paraformaldehyde for 30 min. Following fixation, the cells were stained with the 0.3% Oil Red O solution at room temperature for 1 h. The excess Oil Red O dye was rinsed off with 60% isopropanol for 10 s, followed by a rinse with PBS. Finally, the staining characteristics and morphology of granulosa cells (GCs) were observed under a microscope, and images were captured.

### 2.8. BODIPY Staining of Lipids in GCs

First, the cell samples were washed 2–3 times with PBS to remove the culture medium. Next, 4% paraformaldehyde solution was added to the cell sample to fix them at room temperature for 20–30 min. After fixation, the cell samples were washed 2–3 times with PBS. Then, the BODIPY staining solution was added, and the cell samples were incubated in the staining solution for 10–30 min. The cell samples were washed with PBS 2–3 times to remove unbound staining solution. Subsequently, the DAPI staining solution was added and was incubated at room temperature for 5 min. Finally, the cell sample was observed under a fluorescence microscope using appropriate fluorescence filters to observe BODIPY and DAPI staining signals. For further analysis and processing of the images, the ImageJ software package was utilized.

### 2.9. Detection of Triglycerides (TG) and Cholesterol (CH) in GCs

After 48 h of treatment, cells were collected using RIPA buffer to determine TG and CH content. The ELISA kit instructions (Hengyuan, Shanghai, China) were followed by adding the standard and sample to different wells. Subsequently, the kit’s enzyme-labeled antibody and detection antibody were added. To remove the unbound protein and antibody, the wells were washed with the wash buffer provided by the kit or PBS. Following that, the substrate provided by the kit was added to react with the enzyme-labeled antibody. Finally, the chemical reaction was stopped with the stop solution provided by the kit. Using an ELISA reader at 450 nm OD, the absorbance value was measured, and the concentration of TG and CH in the sample was calculated by drawing a standard curve based on the standard sample.

### 2.10. Lipid-Related Gene Expression Detection

RNA from the cell was extracted according to the instructions using the cell/tissue Total RNA extraction kit (RC101-01 produced by Vazyme Biotechnology Co., Ltd., Nanjing, China). An amount of 2 µL RNA solution was mixed with 0.5 µL 6 × RNA loading buffer and then detected by 1.5% agarose-gel electrophoresis. 1 µL Total RNA was taken, and the concentration and OD (A) values at 280 nm and 260 nm were determined in a nucleic acid protein analyzer. The RNAs with A260/A280 values between 1.8 and 2.0 and the band brightness ratio of 28S: 18S ribosomal RNAs should be about 2:1, and the RNAs with very light 5S bands were used for subsequent reverse transcription tests. The cDNA was synthesized according to the instructions of the reverse transcription kit HiScript III RT SuperMix for qPCR (+gDNA wiper) (R323-01 produced by Vazyme Biotechnology Co., Ltd.). Primer 5.0 was used to design the primers (Table 2). *ACTB* was used as housekeeping gene. The qPCR reaction systems are as follows: 2 × Taq Pro Universal SYBR qPCR Master Mix (Vazyme, Nanjing, China) 10.0 μL, PCR Forward Primer (10 mM) 0.4 μL, PCR Reverse Primer (10 mM) 10.4 μL, cDNA 2 μL, ddH20 7.2 μL. The qPCR amplification conditions are as follows: 95 °C for 30 s; 40 cycles of 95 °C for 5 s and 60 °C for 30 s. The 2^−ΔΔCT^ method was used for normalization of the qPCR results, after which the normalized data were used for statistical analysis, and *p* < 0.05 was considered significantly different.

### 2.11. Statistical Analysis

Experiments for each group were repeated at least three times, and the data are presented as means ± SEM. The statistical differences between groups were analyzed using the T-test analysis method in PRISM. A *p*-value < 0.05 was considered statistically significant, while a *p*-value < 0.01 was considered highly significant.

## 3. Results

### 3.1. Cloning and Sequence Analysis of Goose ESR1

We cloned and obtained the 1866 bp full-length CDS region of the *ESR1* gene in Sichuan white geese. The nucleotide and amino acid homology between different species were shown in Table 3, and the amino acid conservation in poultry is above 98%. Compared with *Danio rerio*, both nucleic acid and amino acid homology are around 50%. Through domain prediction, it was found that 74–207 of the amino acids encoded by the goose *ESR1* gene were estrogen receptor domains (Oest reply), 206–287 were estrogen DNA-binding domains (NR_DBD_ER) composed of two C4 type zinc finger, 336–573 were hormone-activated ligand-binding domains (NR_LBD_ER), and 578–621 were estrogen-type nuclear receptor terminal C-terminal domains (ESR1_C). The results of the evolutionary tree show that the goose *ESR1* gene has the closest relationship with the *Anas platyrhynchos*, which is also a waterfowl, and then converges into the same branch with the *Gallus gallus*, with the farthest relationship with the *Danio rerio*.

### 3.2. Differential Expression of ESR1 in the Granulosa Layer of Follicles at Different Developmental Stages

**Figure 1:**
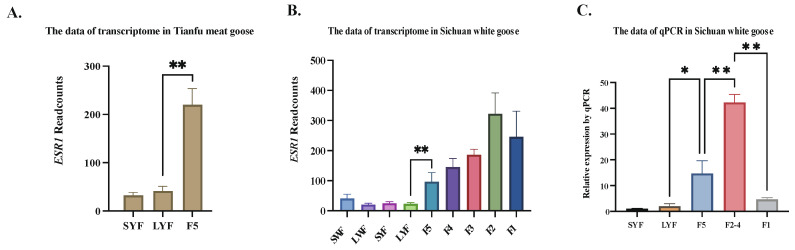
Expression trend of *ESR1* in granulosa layers at different breeds. (**A**) Read counts of *ESR1* in Tianfu meat goose granulosa layers by transcriptome data (N = 3). (**B**) Read counts of *ESR1* in Sichuan white goose granulosa layers by transcriptome data (N = 3). (**C**) Relative expression of *ESR1* in Sichuan white goose granulosa layers by qPCR (N = 3). Note: * represents *p* < 0.05, ** represents *p* < 0.01. Data are shown as Mean ± SEM. SWF: small white follicle; LWF: large white follicle.

As shown in Figure 1, the expression of *ESR1* exhibited a notable increase in the granulosa layers from LYF to F5 follicles development stage in Tianfu meat goose. Similarly, in the transcriptome data of Sichuan white geese, significant changes in *ESR1* expression were observed only during the follicular development stage from LYF to F5. Moreover, the expression of *ESR1* increased in the granulosa layers from the F5 to F2 follicles development stage, while it decreased from F2 to F1.

### 3.3. Effect of ESR1 on Lipid Metabolism of phGCs

**Figure 2:**
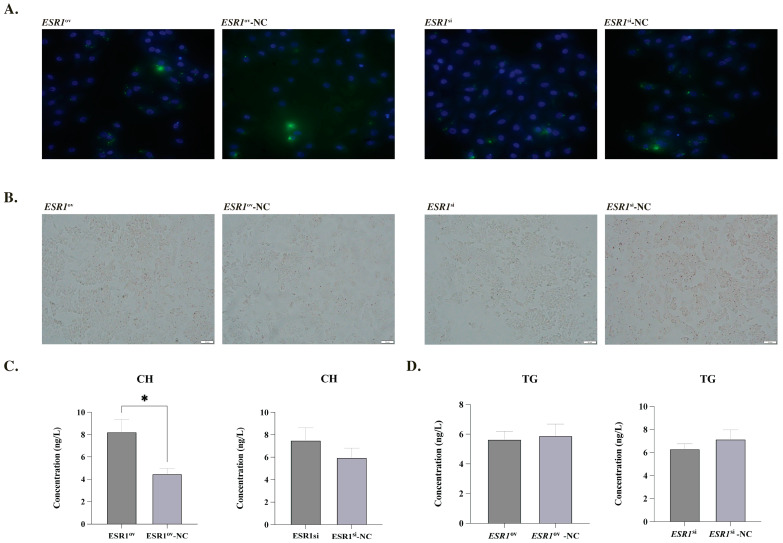
Effect of *ESR1* on lipid metabolism of phGCs. (**A**) Morphological characteristics of LDs in phGCs detection by BODIPY (green) and DAPI (blue) staining. (**B**) Morphological characteristics of LDs in phGCs detection by oil-red O staining. (**C**) Effect of *ESR1* on CH secretion of phGCs (N = 4). (**D**) Effect of *ESR1* on TG secretion of phGCs (N = 4). Note: * represents *p* < 0.05. Data are shown as Mean ± SEM.

The expression of *ESR1* in phGCs was significantly higher in the *ESR1*^ov^ group than in the *ESR1*^ov^-NC group, with a fold change of 142.02. Conversely, the *ESR1*^si^ group had only 46.8% of the expression level of *ESR1* compared to the *ESR1*^si^-NC group in phGCs. The staining results of Oil Red O and BODIPY indicated that there we no significant changes in the lipid droplet (LDs) content in the phGCs after treatment with *ESR1* (Figure 2A,B). ELISA results showed that the CH content in phGCs of the *ESR1*^ov^ group was significantly higher than that of the *ESR1*^ov^-NC group (Figure 2C). However, there was no significant changes in CH content in phGCs between the *ESR1*^si^ and *ESR1*^si^-NC groups (Figure 2C). Meanwhile, there was no significant change in the content of TG in phGC, whether it was in terms of overexpression or interference of *ESR1*. (Figure 2D). Additionally, the expression of genes *ACCα, FASN, DGAT2, PPARα, PPARγ, APOB, SCD, CPT,* and *ATGL* did not change between the two comparisons (*ESR1*^ov^ and *ESR1*^ov^-NC, *ESR1*^si^ and *ESR1*^si^-NC) (Supplementary Figure S1).

### 3.4. Effect of ESR1 on Lipid Metabolism of poGCs

**Figure 3:**
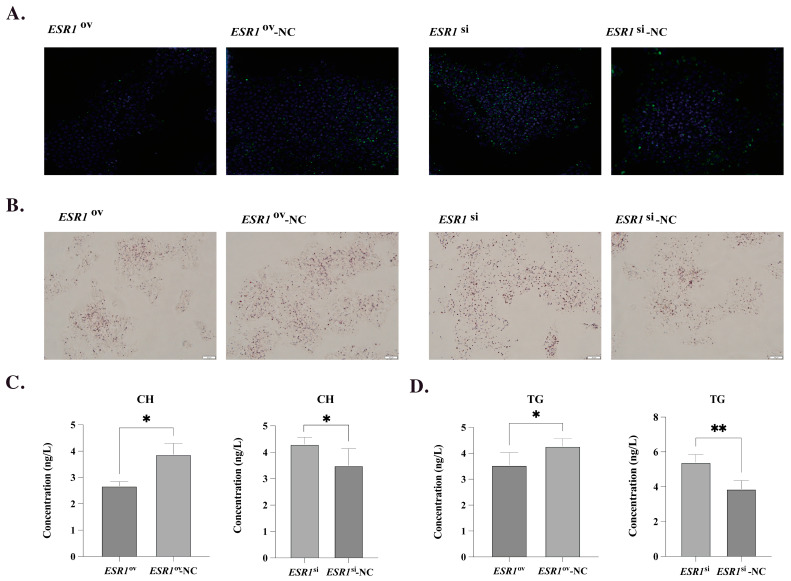
Effect of ESR1 on lipid metabolism of poGCs. (**A**) Morphological characteristics of LDs in poGCs detection by BODIPY (green) and DAPI (blue) staining. (**B**) Morphological characteristics of LDs in poGCs detection by oil-red O staining. (**C**) Effect of *ESR1* on CH secretion of poGCs (N = 4). (**D**) Effect of *ESR1* on TG secretion of poGCs (N = 4). Note: * represents *p* < 0.05, ** represents *p* < 0.01. Data are shown as Mean ± SEM.

The expression of *ESR1* in poGCs was significantly higher in the *ESR1*^ov^ group than in the *ESR1*^ov^-NC group, with a fold change of 176.62. Conversely, the *ESR1*^si^ group had only 74.5% of the expression level of *ESR1* compared to the *ESR1*^si^-NC group in poGCs. The staining results of BODIPY and DAPI showed that overexpression of *ESR1* in poGCs can decrease lipid droplets deposition, while interference of *ESR1* can significantly increase LDs deposition (Figure 3A). Consistently with this, the staining results of oil-red O also showed that the overexpression of *ESR1* could show less LDs deposition, and the interference of *ESR1* showed more LDs (Figure 3B). Moreover, the results of ELISA showed that the interference of *ESR1* could significantly increase the content of CH and TG in poGCs, while the overexpression of *ESR1* could reduce the content of CH and TG (Figure 3C,D).

### 3.5. Expression Profiles of Lipid Metabolism-Related Marker Genes in poGCs

**Figure 4:**
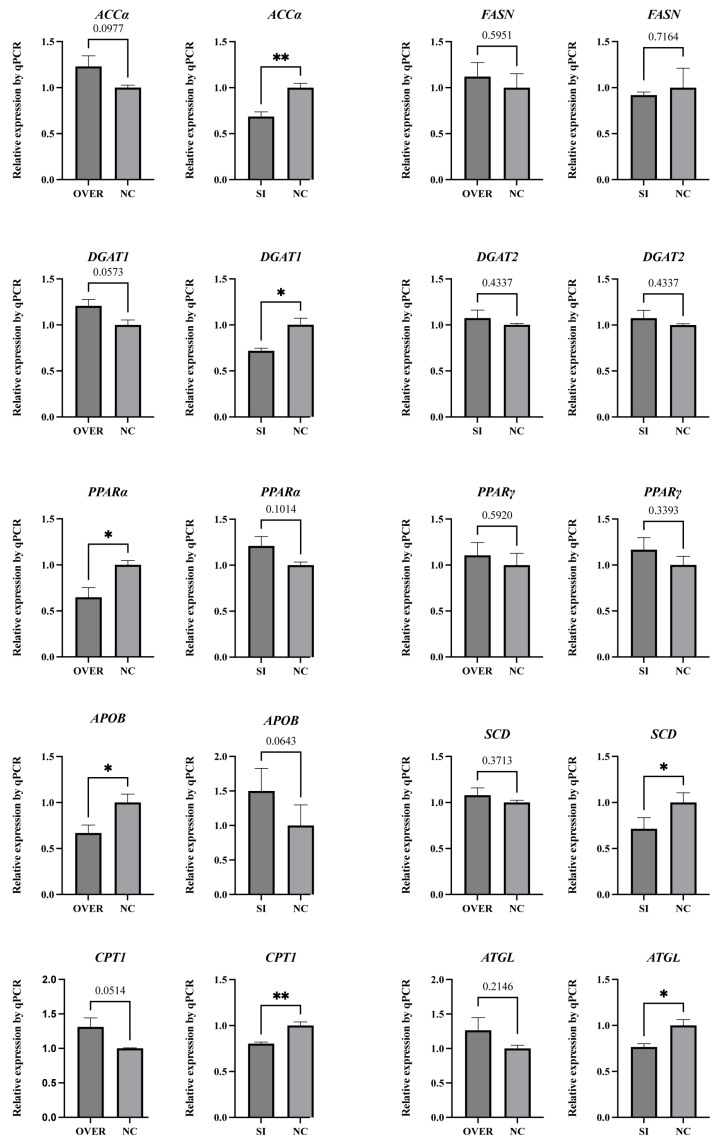
Expression patterns of genes involved in lipid metabolism in poGCs (N = 4). Note: * represents *p* < 0.05, ** represents *p* < 0.01. Data are shown as Mean ± SEM.

Regardless of interference or overexpression of *ESR1* in poGC, the expression of *FASN, DGAT2,* and *PPARγ* did not show significant changes (Figure 4). Compared with the *ESR1*^si^-NC group, the expression of *ACCα*, *DGAT1*, *SCD*, *CPT1*, and *ATGL* was significantly downregulated in the *ESR1*^si^ group. Meanwhile, the expression of *APOB* and *PPARα* in the *ESR1*^ov^ group was significantly lower than that of the *ESR1*^ov^-NC group.

## 4. Discussion

Previous studies have indicated that lipid accumulation in poGCs is higher compared to phGCs [2]. In line with these findings, our study observed significant differences in the expression of *ESR1* in the granular layers between SWF and F5, as well as at F5 and F2–4, and F2–4 and F1. These findings suggest the involvement of *ESR1* in lipid deposition during follicle development. Moreover, it has been reported that phGCs predominantly express genes associated with lipid synthesis and oxidation, while poGCs predominantly express genes related to lipid migration and deposition [2]. Our experimental results further support the notion that *ESR1* primarily influences lipid metabolism in poGCs, indicating its regulatory role in the functions of lipid migration and deposition in these cells.

The LDs are dynamic organelles known for their ability to store neutral lipids and participate in a range of physiological processes [20]. LDs are present in nearly all mammalian follicles and are recognized as an important energy source for oocyte maturation [21,22]. Our experimental results demonstrate that *ESR1* can inhibitor LDs synthesis in goose poGCs, which could be a pathway for its involvement in the development of goose follicles and regulation of egg production.

To further explore the molecular pathway through which *ESR1* participates in lipid metabolism in goose granulosa cells, we conducted qRT-PCR analysis on 10 marker genes associated with lipid metabolism. PPARα and PPARγ are key transcription factors involved in lipid metabolism [23]. *PPARα* promotes the uptake, esterification, and transportation of cellular fatty acids, and it also regulates lipoprotein metabolism genes [24]. Apart from *PPARα*, another gene related to lipoprotein tissue related, *APOB*, showed a significantly downregulation upon *ESR1* overexpression of [25]. Polymorphisms in *ESR1* have been confirmed to be closely associated with human lipoprotein levels [26,27]. Our results demonstrated that the expression of *PPARα* and *APOB* significantly decreased in poGCs upon *ESR1* overexpression. Lipoproteins consist of various lipid and protein components, including TG and CH. Their main function is to transport lipids and proteins in the bloodstream and between tissues and cells [28,29]. Based on these findings, we hypothesize that the decreased expression of *PPARα* and *APOB,* resulting from *ESR1* overexpression, contributes to the reduction in LDs, TG, and CH levels.

*ACCα* plays a crucial role in catalyzing the conversion of Acetyl-CoA to Malonyl-CoA, which is the initial and rate-limiting step in de novo fatty acid biosynthesis [30]. The de novo lipogenesis function of goose granulosa cells has been confirmed, indicating that *ESR1* may enhance de novo lipogenesis in these cells [31]. DGAT1 and DGAT2 are considered to be a key enzyme in TG synthesis [32]. Previous studies have shown that *DGAT1* is not as effective as *DGAT2* in promoting lipid accumulation in poGCs [33]. Mammalian experiments have confirmed that *DGAT1* and *DGAT2* can compensate for each other to a large extent in TG storage [34]. These results suggest that, in goose poGCs, *ESR1*-mediated TG storage is mainly mediated by *DGAT1* rather than *DGAT2*. *SCD* acts as a pivotal enzyme in catalyzing the rate-limiting step of monounsaturated fatty acid production. Recent studies have indicated that *SCD* is involved in the development of goose follicles, specifically promoting LDs deposition and CH storage in granulosa cells [35,36].

Despite observing a significant decrease in the expression levels of three genes involved in fatty acid synthesis (*ACCα, DGAT1, and SCD)* in poGCs following interference of *ESR1,* we still found a significant increase in phenotypes (LDs, TG, and CH). This observation may be closely associated with the expression levels of two genes involved in fatty acid degradation *(ATGL and CPT1). ATGL* initiates the hydrolysis of TGs to release free fatty acids [37]. Fatty acid β-oxidation within mitochondria is a crucial pathway for fatty acid catabolism, playing a pivotal role in maintaining energy homeostasis throughout the body. *CPT1* is the gene encoding an important enzyme in this process [38]. Our findings demonstrate that *ESR1* promotes the expression of both *ATGL* and *CPT1*, thus activating the fatty acid decomposition pathway. The process of fatty acid oxidation also generates ATP, providing energy for granulosa cell activities [39]. This mechanism may represent one of the pathways through which *ESR1* participates in the development of goose follicles.

## 5. Conclusions

In conclusion, we successfully cloned and obtained the complete sequence of the CDS region of the goose *ESR1* gene. We further investigated its expression pattern in granulosa cells at different stages of follicular development. Our experiment results confirmed that there were no significant changes in LDs deposition in phGCs following either overexpression or interference of *ESR1*, while LDs deposition in poGCs significantly decreased after the overexpression of *ESR1* and increased significantly after the interference of *ESR1*. Meanwhile, the content of TG and CH in poGCs significantly decreased after the overexpression of *ESR1*, and the content of TG and CH significantly increased after the interference of *ESR1*. Moreover, qPCR results suggest that these phenotypic results may relate to changes in the expression of *ACCα*, *DGAT1*, *SCD*, *CPT1*, *ATGL*, *APOB*, and *PPARα* in poGCs, which are involved in the synthesis and decomposition of fatty acids and TG, as well as the metabolism and assembly of lipoproteins.

## Figures and Tables

**Table 1:** The sequences of siRNA-*ESR1* oligo.

siRNA	Forward Sequence (5′~3′)	Reserve Sequence (5′~3′)
ESR1-siRNA	GCCACCCAUAGUUUAUUCUTT	AGAAUAAACUAUGGGUGGCTT
siRNA-NC	UUCUCCGAACGUGUCACGUTT	ACGUGACACGUUCGGAGAATT

**Table 2:** Primer used in this study.

Primer Name	Forward Sequence (5′-3′)	Reserve Sequence (5′-3′)	Product Size (bp)
*ACTB*	CAACGAGCGGTTCAGGTGT	TGGAGTTGAAGGTGGTCTCG	92
*ESR1*	GAACAATGTCCCACCAAACCCT	TGGATAGGCTCCCTTTCTCGTTA	227
*PPARα*	ATCTATCCCTGGCTTCTCCA	AGCATCCCATCCTTGTTCATT	117
*PPARγ*	CCTCCTTCCCCACCCTATT	CTTGTCCCCACACACACGA	108
*APOB*	CTCAAGCCAACGAAGAAG	AAGCAAGTCAAGGCAAAA	153
*CPT-1*	GTCTCCAAGGCTCCGACAA	GAAGACCCGAATGAAAGTA	193
*SCD1*	GCCATCGGTCCTACAAAGC	AGCCAATGTGGGAGAAGAAA	180
*DGAT1*	CCTGAGGAACTTGGACACG	CAGGGACTGGTGGAACTCG	265
*DGAT2*	CGCCATCATCATCGTGGT	CGTGCCGTAGAGCCAGTTT	113
*ACCα*	TGCCTCCGAGAACCCTAA	AAGACCACTGCCACTCCA	163
*FASN*	TGGGAGTAACACTGATGGC	TCCAGGCTTGATACCACA	109
*ATGL*	TCGCAACCTCTACCGCCTCT	TCCGCACAAGCCTCCATAAGA	300

**Table 3:** Nucleotide and amino acid sequence identity of ESR1 among 7 vertebrate species.

Species	Nucleic Acid Length	Nucleic Acid Homology (%)	Amino Acid Length	Amino Acid Homology (%)
*Anser cygnoides*	1866	100.00	621	100.00
*Gallus gallus*	1770	94.63	589	98.30
*Anas platyrhynchos*	1887	98.55	628	98.55
*Xenopus Laevis*	1758	71.90	585	76.67
*Danio rerio*	1842	56.78	613	52.42
*Homo sapiens*	1788	75.28	595	78.91
*Mus musculus*	1800	74.24	599	78.23

## Data Availability

Not applicable.

## Supplementary Materials

The following supporting information can be downloaded at: https://www.mdpi.com/article/10.3390/biology12070962/s1, Figure S1: Expression patterns of genes involved in lipid metabolism in phGCs.
