# Supplementary figures and images for: Estrogen Receptor Gene 1 (ESR1) Mediates Lipid Metabolism in Goose Hierarchical Granulosa Cells Rather than in Pre-Hierarchical Granulosa Cells
Source: Biology (Basel). 2023 Jul 5;12(7):962. doi: 10.3390/biology12070962 (PMC10376489; doi:10.3390/biology12070962)

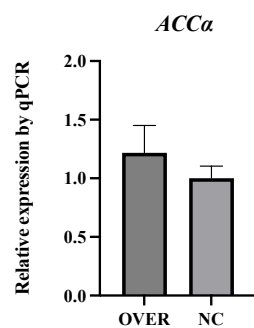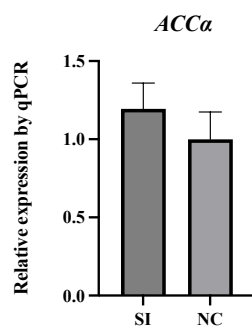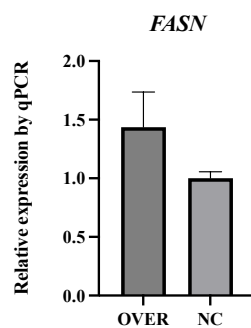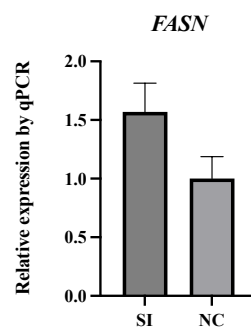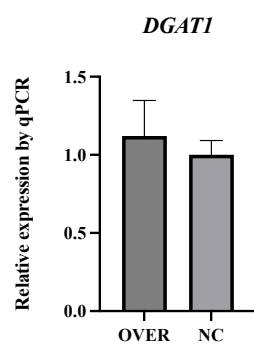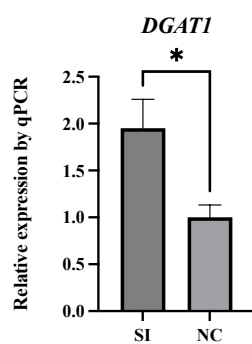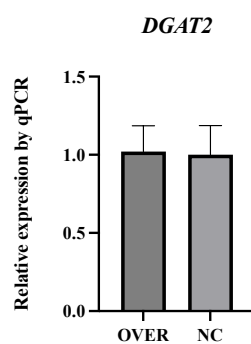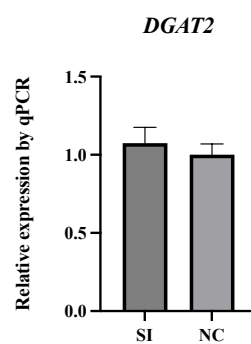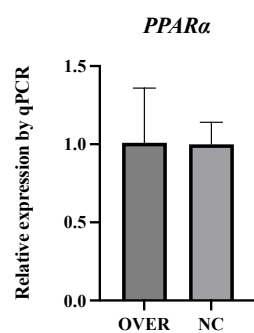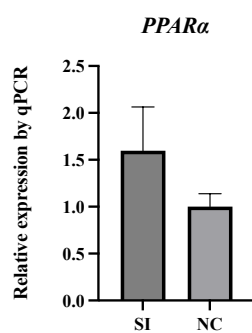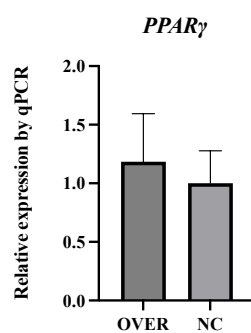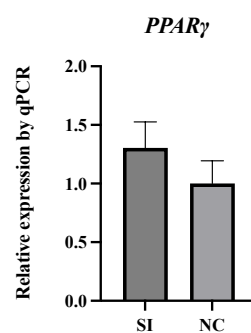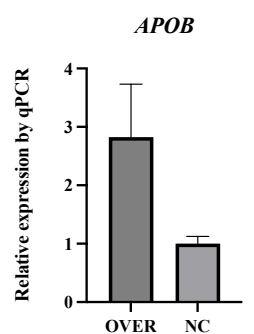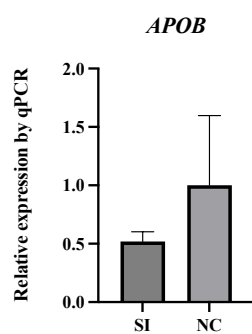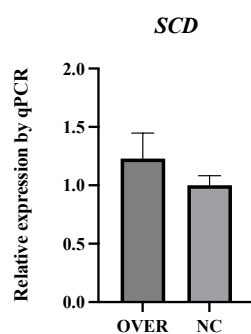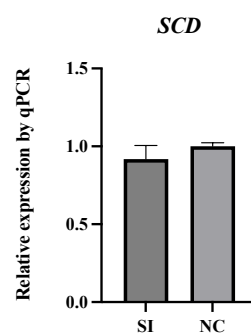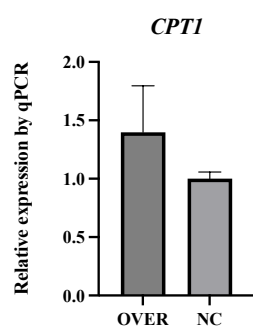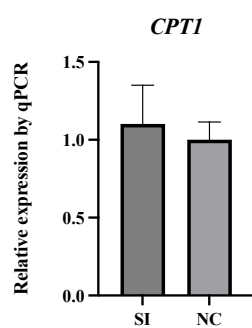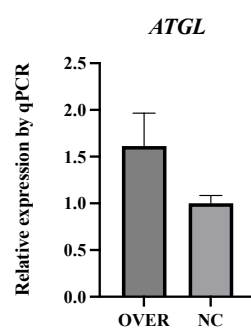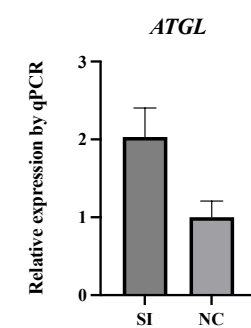

Supplement: Supplementary file 1 [file biology-12-00962-s001.zip › biology-2362774-supplementary.pdf]
